# Improving students' mathematics self-efficacy: A systematic review of intervention studies

**DOI:** 10.3389/fpsyg.2022.986622

**Published:** 2022-09-26

**Authors:** Yusuf F. Zakariya

**Affiliations:** Department of Mathematical Sciences, University of Agder, Kristiansand, Norway

**Keywords:** intervention, statistics, social cognitive theory, performance in mathematics, mathematics self-efficacy

## Abstract

Self-efficacy is an integral part of personal factors that contributes substantially to students' success in mathematics. This review draws on previous intervention studies to identify, describe, and expose underlying mechanisms of interventions that foster mathematics self-efficacy. The findings show that effective mathematics self-efficacy interventions can be categorized into three categories using their underlying mechanisms: those that directly manipulate sources of self-efficacy to foster the construct, and those that either embed self-efficacy features in teaching methods or in learning strategies. Specific examples of interventions that fall in each of these three categories are described including their features and the underlying mechanisms that improve students' mathematics self-efficacy. I argue for the two “most effective” interventions that foster mathematics self-efficacy and their relevance to either pre-university or university students with implications for teaching and learning of mathematics.

## Introduction

### Background

Research on affect in mathematics education is attracting increased attention such that researchers are proposing a unifying theoretical framework for its various constructs. For instance, Hannula (2012) proposed a unified theoretical approach to these constructs within mathematics education research community. Some of the constructs that characterize affect in mathematics education research are attitudes toward mathematics, beliefs in mathematics, mathematics anxiety, mathematics emotions, mathematics hate, mathematics joy, and mathematics self-efficacy. Students' mathematics self-efficacy is a crucial construct that has historic significance to affect in mathematics education research (Hannula, 2012). Its origin is traceable to Bandura's social cognitive theory that sees human functioning as an emergence of a dynamic interaction system between personal (e.g., self-efficacy), behavioral (e.g., use of effective approaches to learning), and environmental (e.g., teacher's feedback) determinants (Bandura, 1997, 2012). That is, students with high self-efficacy are quick to engage in behavioral activities (e.g., using efficient approaches to learning) that enhance their successful execution of the presented tasks. In turn, quality feedback from teachers or peers is a crucial factor that reinforces self-efficacy (Bandura, 1997). This dynamic interaction between the personal, behavioral, and environmental determinants characterizes human functioning within and outside formal classroom settings (Bandura, 2001).

Self-efficacy is a personal determinant of human functioning is defined as “beliefs in one's capabilities to organize and execute the courses of action required to produce given attainments” (Bandura, 1997, p. 3). Within the context of mathematics learning, mathematics self-efficacy is conceptualized as “a situational specific assessment of an individual's confidence in her or his ability to successfully perform or accomplish a particular [mathematics] task or problem” (Hackett and Betz, 1989, p. 262). Mathematics self-efficacy encompasses students' interpretation of their prior attainments, a self-appraisal of their ability, and a personal estimation of subsequent performance on presented mathematics tasks. It is an important construct that determines students' engagement with mathematics tasks. Some students engage in the tasks they feel confident to solve and avoid tasks they believe are out of their competence level. Mathematics self-efficacy influences students' choices of tasks on which they will expend much effort, it determines students' level of perseverance and the amount of forbearance in difficult situations (Pajares, 1996; Zakariya et al., 2019). As such, mathematics self-efficacy is a self-evaluation of students' competence about the presented mathematics tasks which constitutes an internal drive for the successful completion of the task.

Given the importance of mathematics self-efficacy to students' learning experience, several interventions on the construct are described in literature (e.g., Siegle and McCoach, 2007; Schukajlow et al., 2019), mostly by educational psychologists, as a proxy to improve students' learning outcomes in mathematics. However, few studies provide coherent arguments about which and to what extent interventions enhance students' mathematics self-efficacy. This review attempts to fill this gap by drawing on previous intervention studies to identify, describe, and expose underlying mechanisms of interventions that foster mathematics self-efficacy.

### Task specificity of mathematics self-efficacy

There is an accumulation of both empirical and theoretical evidence that suggests that mathematics self-efficacy is best operationalised and measured using task specific instruments. Researchers argued that the task specificity of mathematics self-efficacy must be duly accounted for to enhance predictive power of the construct (e.g., Bandura and Schunk, 1981; Hackett and Betz, 1989; Pajares and Miller, 1995; Klassen and Usher, 2010; Toland and Usher, 2015). The implication of the task specificity goes beyond the predictive power of mathematics self-efficacy to the development of its measures. Students are more likely to accurately report their convictions to be able to solve mathematics tasks when sample tasks are presented to them than the situation in which sample tasks are not presented. Borgonovi and Pokropek (2019) investigate this fact and found a non-trivial link between task exposure and mathematics self-efficacy among secondary school students. This task-specific measure of mathematics self-efficacy makes it different in conceptualization as well as in measurement from a related construct called mathematics self-concept. The formal is task-specific while the latter is the belief of self-worth associated with one's perceived competence without any reference to neither specific situation nor to specific task (Pajares and Miller, 1994). As such, several measures of mathematics self-efficacy are tailored toward specific tasks in mathematics rather than general mathematics (Hackett and Betz, 1989; Pajares and Miller, 1995; Kranzler and Pajares, 1997; Zakariya et al., 2019). For instance, the calculus self-efficacy inventory developed by Zakariya (2019) requires respondents to rate their confidence to solve some presented calculus exam-like tasks on a scale of 0 to 100.

### Sources of mathematics self-efficacy

Apart from the conceptualization, task specificity, and measures of mathematics self-efficacy, a sizeable number of studies are reported on the sources of mathematics self-efficacy. Drawing on Bandura's social cognitive theory, four sources of mathematics self-efficacy are theorized, investigated, and measured in literature (e.g., Lent et al., 1991; Usher and Pajares, 2009; Gao, 2019). Students build their self-efficacy based on interpretations of events that emanate from four sources: “mastery experience,” “verbal/social persuasions,” “physiological or affective states,” and “vicarious experience” (Bandura, 2012). The mastery experience encapsulates students' interpretations of their previous academic attainments in mathematics. It is the strongest source of mathematics self-efficacy (Zientek et al., 2019). Success reinforces self-efficacy while failure mars it. Students that accomplish a mathematics task, especially a difficult task for others, interpret their success in a positive way such that the interpretation elevates their judgement of competence in mathematics. In contrast, students' interpretation of failures on mathematics tasks tend to lower the judgement of their competence in mathematics (Usher and Pajares, 2009). It is crucial to remark that students' interpretations of the same academic achievement (e.g., same grades) may differ, and so does the impact of such achievement on individual's mathematics self-efficacy. Thus, individual interpretation of mastery experience is pertinent to mathematics self-efficacy rather than the objective grade in itself (Lopez et al., 1997).

Social persuasion is a source of mathematics self-efficacy that students make while listening to verbal persuasion from other people. The timely encouragement from teachers, parents, peers, and more proficient adults are likely to foster students' confidence when dealing with challenging situations. On the other hand, negative remarks from others undermine students' mathematics self-efficacy in the face of obstacles. In fact, the influence of social persuasion on mathematics self-efficacy is more pronounced in weakening self-efficacy rather than bolstering it (Usher and Pajares, 2009). Empirical evidence also suggests that social persuasion is a substantive source of mathematics self-efficacy as after mastery experience (Lopez et al., 1997; Yurt, 2014). However, some researchers (e.g., Lau et al., 2018) show that social persuasion predicts self-efficacy better than mastery experience.

The physiological or affective source of mathematics self-efficacy entails the self-evaluation of competence on mathematics tasks that draws on varying levels of students' emotions such as anxiety, mood, attitudes, and physiological arousals such as burnout, fatigue, and stress. Students who feel secured, relaxed, and emotionally stable while engaging in mathematics activity tend to judge their competence on the mathematics activity very highly. In contrast, emotional instability, burnout, fatigue, and stress play crucial role in weakening students' evaluation of their competence on mathematics tasks. As stated by Usher and Pajares (2009) “increasing students' physical and emotional wellbeing and reducing negative emotional states strengthens self-efficacy” (p. 90).

The vicarious experience is a source of self-efficacy that relates to students' interpretation of others' experience (Matsui et al., 1990). Observing comparable others who succeed in completing a mathematics task is a crucial source of mathematics self-efficacy (Usher and Pajares, 2009). Students can draw on the successes or failures of their peers, colleagues, and comparable others in a mathematics task to make self-evaluation of their competence on the task (Usher and Pajares, 2009). Among the four sources of mathematics self-efficacy, evidence shows that the vicarious experience exhibits the least influence on self-efficacy (Lopez et al., 1997; Loo and Choy, 2013). However, some researchers (e.g., Usher and Pajares, 2008) argued that the inclusion of either peers or adults and not both in measures of vicarious experience can be ascribed to its least rank among the sources of mathematics self-efficacy. Interestingly, all the four sources of mathematics self-efficacy predict, though at varying strengths, not only the self-efficacy but also students' achievements in mathematics (Usher and Pajares, 2008; Zientek et al., 2019).

### Self-efficacy and other affective factors

Self-efficacy predicts and it is predicted by factors such as mathematics self-concept, mathematics anxiety, interest, emotional support, motivational processes, and students' approaches to learning (Pajares and Miller, 1994; Lopez et al., 1997; Akin and Kurbanoglu, 2011; Zakariya et al., 2020). There is a substantial correlation between self-efficacy and mathematics self-concept, interest, and perceived usefulness of mathematics (Pajares and Miller, 1994; Lopez et al., 1997). Also, previous studies (e.g., Akin and Kurbanoglu, 2011; Rozgonjuk et al., 2020) show that there is a bidirectional relationship between mathematics anxiety and mathematics self-efficacy. That is, students with high self-efficacy tends to exhibit low mathematics anxiety. In turn, students with high mathematics anxiety are associated with low self-efficacy on mathematics tasks. More so, Skaalvik et al. (2015) show that students' perception of emotional support received from teachers predicts mathematics self-efficacy which in turn predicts motivational processes such as effort expends on mathematics tasks, persistence on difficult mathematics problems, intrinsic motivation, and help seeking disposition. On the other hand, Özcan and Eren Gümüş (2019) show that mathematics motivation predicts mathematics self-efficacy which in turn predicts retrospective metacognitive experience i.e., students' narrative of their metacognitive activities after solving a mathematics task.

Evidence shows that mathematics self-efficacy is substantially related to students' approaches to learning mathematics (e.g., Diseth, 2011; Ardura and Galán, 2019; Zakariya et al., 2020). In fact, Zakariya et al. (2020) show that there is a potential causal relationship between mathematics self-efficacy and approaches to learning first-year calculus course. That is, students with high mathematics self-efficacy adopt deep approaches to learning first-year calculus while those with low mathematics self-efficacy adopt surface approaches to learning the course (Zakariya et al., 2020). Thus, mathematics self-efficacy influences students' processes and strategies with which they study for mathematics. An in-depth understanding of the underlying mechanism of factors that affect and are affected by mathematics self-efficacy are crucial for developing interventions. As such, the relationship between mathematics self-efficacy and some personal factors has direct implications for this review.

### Self-efficacy and performance in mathematics

Several researchers have thoroughly investigated the association between mathematics self-efficacy and students' performance in mathematics. The performance in mathematics, here, means students' examination scores or grades in mathematics courses that they followed. Their findings show that mathematics self-efficacy predicts performance better than mathematics anxiety, mathematics self-concept, mental ability, prior mathematics knowledge, and perceived utility of mathematics (Pajares and Miller, 1994; Pajares and Kranzler, 1995; Özcan and Eren Gümüş, 2019). Also, mathematics self-efficacy predicts performance in mathematics better than intelligence test scores, personality traits (i.e., agreeableness, conscientiousness, emotional instability, extraversion, and openness), and self-esteem (Zuffianò et al., 2013). There seems to be a consensus that mathematics self-efficacy has a substantial positive association with students' performance in mathematics (Pajares and Miller, 1995; Yurt, 2014; Roick and Ringeisen, 2018; Zakariya, 2021). That is, high mathematics self-efficacy is associated with high performance in mathematics while low mathematics self-efficacy is associated with poor performance in mathematics. Going a step further, Zakariya (2021) uses an innovative instrumental variable approach of structural equation modeling to show that students' mathematics self-efficacy on mathematics tasks has a causal relation with students' performance in mathematics.

It is important to mention that the crux of the matter of improving mathematics self-efficacy is to serve as a proxy to improve students' performance in mathematics. As the literature suggests, mathematics self-efficacy does not only predict students' performance in mathematics but also has a potential causal relationship with performance (Pajares and Miller, 1995; Yurt, 2014; Roick and Ringeisen, 2018; Zakariya, 2021). A pedagogical implication of this relationship is the opportunity made available to mathematics teachers to improve students' performance in mathematics through reinforcement of mathematics self-efficacy. On the part of the students, a high sense of mathematics self-efficacy mitigates their mathematics anxiety and thereby reduces their risk of failure in mathematics (Rozgonjuk et al., 2020; Zakariya, 2021). On the one hand, these connections between mathematics self-efficacy and the final learning outcomes (e.g., students' performance in mathematics and risks of failure in mathematics) lay more credence to the utility of interventions that reinforce self-efficacy among students following a mathematics course. On the other hand, these connections also buttress the importance of a systematic review of such interventions.

### The research aims

This article reports a systematic review of intervention studies that is aimed at improving students' mathematics self-efficacy. In the preceding sections, I presented the theoretical structures of mathematics self-efficacy, its sources, its crucial contributions to other personal factors and students' performance in mathematics. The discussion in each of these sections points to the significance of altering mathematics self-efficacy such that students' performance in mathematics can improve. Thus, studies are that aimed at altering students' mathematics self-efficacy are crucial not only to students' well-being but also to improved performance in mathematics. Therefore, the purpose of this review is to provide an integrative view of previous intervention studies on mathematics self-efficacy. Therein, I am addressing the following questions:

- What are the interventions that enhance self-efficacy and their underlying mechanisms?
- Which of these interventions has the largest effect on self-efficacy?

The researcher believes that attempts to address these research questions will expose the state of the art on interventions that reinforce mathematics self-efficacy. Since knowledge progression is usually built on existing knowledge, it becomes prudent to critically examine the existing knowledge. A review, analysis, and synthesis of relevant literature will reveal the state of the art as it concerns interventions on mathematics self-efficacy. Surprisingly, there is little research with this intention. Admittedly, there is an earlier review of literature on self-efficacy by Bartimote-Aufflick et al. (2015). However, they restrict the focus of their review to higher education students, and they consider self-efficacy without a particular reference to mathematics learning. Given that mathematics self-efficacy is task specific as pointed out in the background section, the present review will provide more relevant details to mathematics learning than the one by Bartimote-Aufflick et al. (2015). More so, researchers, mathematics teachers, mathematics course coordinators, and other stakeholders will benefit from the findings of this review. Such benefits would be in the form of what to do, how to do it, and to what extent do interventions alter mathematics self-efficacy for improved performance in mathematics.

## Methods

### Review process

**Figure 1:**
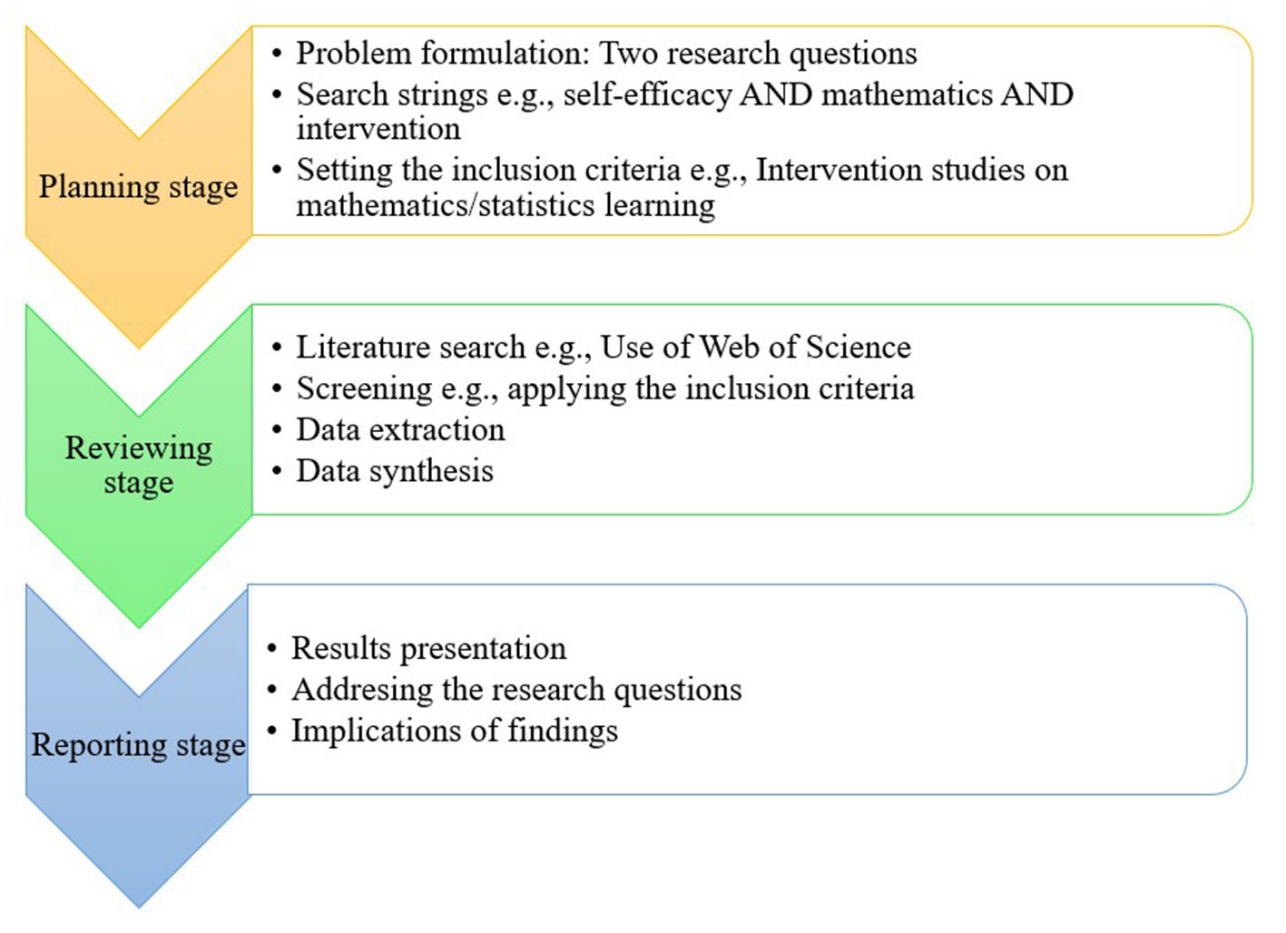
Specifics of stages of the review process.

This review followed a framework proposed by Kitchenham and Stuart (2007) and developed further by Xiao and Watson (2017). In this framework, three main stages were identified: planning, conducting, and reporting the review. Figure 1 shows the specifics of each of these stages as they relate to the present review.

### Planning stage

The planning stage of this review started by defining the purpose and scope of the review. The purpose was to provide an integrative view of previous intervention studies on mathematics self-efficacy. This purpose led to the formulation of two research questions. As such, I defined “self-efficacy,” “mathematics,” “statistics,” “mathematics self-efficacy,” and “intervention” as keywords for conducting the literature search. I set the criteria for inclusion of studies to be only intervention studies that focus on self-efficacy of students learning either mathematics or statistics. I restricted the scope of this review to mathematics/statistics learning because of the task-specificity of the construct. Further, I included in this review all types of experimental studies e.g., one group pre-test/post-test experiments, quasi-experiments, and randomized control trials that are available in English language.

### Reviewing stage

In the reviewing stage, I searched two main literature databases i.e., Web of Science and ERIC for articles published between 1995–2021, using a combination of keywords that were defined in the planning stage. These two databases were used because of their popularity in education research and the quality of articles they indexed. The search on Web of Science returned 220 publications while the search on ERIC returned 195 publications with a substantial overlap at the time of the literature search. I reviewed the titles and read the abstracts of these publications together with some colleagues using the criteria for inclusion as presented in the previous section. Most of the publications were based on measures of self-efficacy, relationships between self-efficacy with other personal factors, and performance. These publications were excluded from the sample of this review because they were not intervention studies. Also, few of the publications were theses, dissertations, and previously presented conference papers of current journal articles. These publications were excluded from the sample as well because I preferred peer-reviewed articles to the evolving publications in theses and conference papers. This screening process led to 17 peer-reviewed journal articles. Then, we checked the references of each of these 17 articles for relevant studies. An additional four articles were identified through this snowballing approach to make 21 peer-reviewed journal articles. We read each of the 21 articles extensively to assess the quality and extract relevant data for this review. The extracted data were then categorized, synthesized, and analyzed to make a coherent argument for this review.

### Reporting stage

This is the last stage of the review process. I assimilated the results such that the two research questions were addressed. Further, I discussed some implications of the findings for researchers, mathematics teachers, mathematics course coordinators, and other stakeholders that are involved in the teaching and learning of mathematics.

## Results and discussion

### Interventions that enhance mathematics self-efficacy

To identify interventions that enhance mathematics self-efficacy, and as such to address the first research question, I provide a summary of findings from the 21 reviewed articles. Table 1 gives a summary of these results. One can observe from Table 1 that 16 of the 21 reviewed studies attributed significant increase in mathematics self-efficacy to the interventions reported. However, if we exclude single-group experiments and case-study experiments due to their high susceptibility to internal and external validity threats (Bryman, 2016) then 11 studies remain which interventions are worthy of further discussion. These studies are marked with asterisks (^*^) in Table 1 and in the reference list. Also, further reading suggests that the study by Huang et al. (2020) is an improvement on their two earlier studies (Huang, 2017; Huang and Mayer, 2018). As such, only the latest study is discussed in this review. There are then nine studies, four of which are quasi-experimental research (Ramdass and Zimmerman, 2008; Bonne and Johnston, 2016; Kandil and Işiksal-Bostan, 2019; Kohen et al., 2019) and the remaining five studies are randomized controlled experiments (Luzzo et al., 1999; Siegle and McCoach, 2007; Cordero et al., 2010; Brisson et al., 2017; Huang et al., 2020).

**Table 1:** Summary of findings of the reviewed articles.

**Author**	***N***	**Type**	**Group**	**Intervention**	**Treatment**	**Mechanism**	**Intervention duration**	**Outcome**	**Effect size**
Bartsch et al. (2012)	39	QE	2	Peer model presentation	E (20) - peer model presentation C (19) - writing about successful students in the course	Vicarious experience	A 10-minute presentation	Marginal increase in self-efficacy	*d* = 0.45
*Bonne and Johnston (2016)	91	QE	2	Pedagogical strategies	E (41)- Pedagogical strategies C (50) - Not reported	Instructional method	3 months	Significant increase in self-efficacy	*d* = 0.39
*Brisson et al. (2017)^se^	1,916	RCE	3	Mathematics relevance intervention	E1 (561)-quotations (self-reflection on the relevance of mathematics by reading interview quotations from young adults) E2 (720) - text (original arguments for the relevance of mathematics) C (635) - No treatment	Vicarious experience	6 weeks	significant increase in self-efficacy by E1	β = 0.16
Cuenca-Carlino et al. (2016)	6	Case	3	Self-regulated strategy development (SRSD) model of instruction	SRSD instruction	Instructional method	12 weeks	Significant increase in self-efficacy	Nil
*Cordero et al. (2010) ^se^	99	RCE	2	Performance accomplishment plus belief perseverance	E (51): Performance accomplishment plus belief perseverance experimental group C (48): Performance accomplishment	Self-persuasion	27 min	Significant increase in self-efficacy	
Czocher et al. (2019)	90	QE	1	Modeling competition	Modeling competition	Mastery experience	Not reported	Significant gain in self-efficacy	*d* = 0.55
Falco et al. (2010) ^se^	153	QE	2	Curriculum design principles	E (79): Curriculum design principle instruction C (74): Regular mathematics instruction	Instructional method	9 weeks	Improved self-efficacy only for girls	β = 0.25
Getachew and Asfawossen (2016)	123	QE	2	Instructional method	E (63): Taught using the specially designed instructional strategies C (60): Taught using the usual instructional method	Mastery, vicarious, verbal, and emotional experiences	4 weeks	No significant difference is self-efficacy	Not reported
Grothérus et al. (2019)	22	FG	1	Formative scaffolding programme (FSP)	Engagement in FSP	Self-regulation and feedback	Not reported	Positive impact on self-efficacy	Not reported
Hanlon and Schneider (1999)	17	FG	1	Summer camp that includes use of goal-setting and self-monitoring techniques	Engagement in self-efficacy instruction summer camp	Instructional method	5 weeks	Significant increase in self-efficacy	Not reported
*Huang (2017)	116	QE	4	A computer-based example-based learning	E1: Standard worked examples E2: Erroneous worked examples E3: Masterly modeling example E4: Peer coping modeling example	Instructional method	1 h and 30 min	E4 has most significant gain in self-efficacy	Not reported
*Huang and Mayer (2018)	142	RCE	2	Adding self-efficacy features to computer-based example-based learning	E (71): Self-efficacy features integrated into the example-problem situation C (71): Worked example-problem situation practice activity	Sources of self-efficacy	57 minutes	Significant increase in self-efficacy	*d* = 0.44
*Huang et al. (2020)	279	RCE	6	Adding self-efficacy features to computer-based example-based learning	E1 (48): Anxiety coping strategies E2 (49): Modeling example E3 (49): Mental practice group E4(45): Effort feedback group E5 (47): Integrated strategies group C (41): Control group	Sources of self-efficacy	1 h	Significant higher self-efficacy of E5-group than C-group has the most	*d* = 0.71
*Kandil and Işiksal-Bostan (2019)	48	QE	2	Inquiry-based instruction enriched with Origami	E (23): Inquiry-based instruction C (25): Regular instruction	Instructional method	3 weeks	Significant increase in self-efficacy	Not reported
*Kohen et al. (2019)	11	QE	2	Instructional method	E1 (58): Dynamic visualization using GeoAlgbra E2 (53): Static visualization using the board or textbooks	Instructional method	5 weeks	Significant higher self-efficacy in E1 than in E2	Not reported
*Luzzo et al. (1999) ^se^	94	RCE	4	Performance accomplishment and vicarious learning experiences	E1 (22): Vicarious learning E2 (22): Performance accomplishment E3 (26): Combine 1 and 2 C (24): No treatment	Sources of self-efficacy	Less than one hour	Significant increase in self-efficacy of E2 and E3	*d* = 0.51 (for E2)
*Ramdass and Zimmerman (2008)	42	QE	2	Learning strategies	E (21): Step-by-step solution strategy plus self-correcting strategy C (21): Step-by-step solution strategy	Learning strategy	50 min	Significant increase in self-efficacy	Not reported
Ritzhaupt et al. (2011)	225	QE	1	Educational game	Pre-algebra and algebra game	Instructional method	16 weeks	Significant increase in self-efficacy	
Samuel and Warner (2019)	40	QE	2,1	Mindfulness and growth mindset	**First experiment (FE)** E (20): Mindfulness/growth mindset ideas embedded in instructional method C (20): Normal instructional method **Second experiment (SE)** Mindfulness/growth mindset ideas embedded in instructional method	Instructional method	12 weeks	No significant increase is self-efficacy of FE Significant increase in self-efficacy of SE	r=-0.57, and 0.48
Schukajlow et al. (2019)	304	QE	3	Constructing multiple solutions	E1: Two mathematical solution methods E2: One solution1 E3: One solution2	Instructional method	56 min	No significant increase in self-efficacy	Nil
*Siegle and McCoach (2007)	872	RCE	2	Self-efficacy teacher training	E (430): Taught by teachers who received self-efficacy training C (442): Taught by teachers who do not receive self-efficacy training	Sources of self-efficacy	4 weeks	Significant increase in self-efficacy	*d* = 0.46

RCE-randomized control experiment, QE-quasi experimental, FCE-focused group experiment, d-Cohen's effect size, η^2^-partial eta squared, β-standardized regression coefficient, and r-nonparametric correlation coefficient. The four studies that reported sustained effects of their interventions are marked with the suffix

se attached to the authors' names.

Content analysis of the nine intervention studies shows that the interventions can be grouped and discussed according to their underlying mechanisms. Based on the findings of this analysis, three categories emerge: Interventions based on mathematics self-efficacy sources (IbMSES), instructional-based interventions (IbI), and learning-based interventions (LbI). The criteria for inclusion of a study in the IbMSES category are that the study manipulates at least a source of mathematics self-efficacy, and the source(s) is (are) explicitly stated. As for the IbI and LbI categories, the criteria are that the studies embed mathematics self-efficacy sources/strategies in the teaching and learning of mathematics, respectively. Admittedly, it is difficult to separate learning from teaching. As such, the IbI and LbI categories appear similar but at the same time different.

### Interventions based on self-efficacy sources

These interventions are based on manipulating one or more sources of mathematics self-efficacy such that the students' mathematics self-efficacy can be enhanced. The first intervention is this category is the *mathematics relevance intervention* by Brisson et al. (2017) with an effect size of 0.16 that was sustained over a period of 6 weeks. The most effective treatment group in their experiment, in terms of fostering mathematics self-efficacy, is the quotation condition. Students in the quotation condition engaged in teacher-led presentations that focus on confidence reinforcement and relevance of mathematics to real-life situations. Thereafter, students engage in an in-class self-reflection on relevance of mathematics to daily lives by reading interview quotations from young adults. This intervention is followed-up by two homework short intervention reinforcements that focus on recalling aspect of in-class self-reflection and self-evaluation of arguments about utility of mathematics. The basic mechanism of fostering mathematics self-efficacy in quotation treatment condition lies in using the utility of mathematics to provide vicarious experience to students.

Luzzo et al. (1999) combined performance accomplishment and vicarious experience to design interventions that foster mathematics self-efficacy with an effect size of 0.51 that was sustained over a period of 4 weeks. In a similar manner, Cordero et al. (2010) combined performance accomplishment with belief perseverance to design an intervention that fosters mathematics self-efficacy by with an effect size of 0.09 that was sustained over a period of 6 weeks. The idea behind performance accomplishment intervention in both studies requires students to solve some mathematics problems, mark their answers by themselves, and then rate their accomplishment in attaining a pre-set criterion for success before the experiment. The vicarious experience intervention by Luzzo et al. (1999) requires students to watch a video presentation of a senior colleague(s) that has previously followed the target mathematics course. In the videotaped presentation, the models share their experience while following the course and how the course has helped them in their career aspirations. On the other hand, the belief perseverance intervention by Cordero et al. (2010) requires students to write a proposal that justifies their suitability for a fully-funded scholarship that centers around their belief of success in demanding mathematics activities. The basic mechanisms that foster mathematics self-efficacy in these interventions are self-persuasion and vicarious experience.

### Instructional-based interventions

These are interventions that foster mathematics self-efficacy through manipulations of teaching methods. Inquiry-based instruction enriched with Origami was proved effective in enhancing students' self-efficacy on mathematics tasks (Kandil and Işiksal-Bostan, 2019). Origami has to do with folding papers for instructional purpose with relevance to geometry (Kandil and Işiksal-Bostan, 2019). Kohen et al. (2019) show that incorporating dynamic visualization activity e.g., use of GeoGebra application, into an active instructional method is another effective way to enhance students' mathematics self-efficacy. They taught some students analysis of functions using the dynamic visualization instruction and found that mathematics self-efficacy is improved afterwards. The underlying mechanisms that foster mathematics self-efficacy in these interventions are the additional reinforcement offered by Origami and the dynamic digital software, respectively.

From teachers' perspective to students' enhancement of mathematics self-efficacy, some researchers reported interventions that focus on manipulating teacher professional development programmes or training (Siegle and McCoach, 2007; Bonne and Johnston, 2016). The intervention study by Siegle and McCoach (2007) show that teacher training that centers around goal setting, quality teacher feedback, and peer modeling can foster mathematics self-efficacy. The goal setting involves activities that remind students of their mastery experience. The teacher feedback serves as social persuasion through complimenting students' effort, and the peer modeling provides vicarious experience to the students. In a similar manner, Bonne and Johnston (2016) show how pedagogical strategies can be used to enhance mathematics self-efficacy. Some of these strategies are sharing instructional objectives with students, reminding students of their mastery experience, encouraging students to attribute failure to lack of sufficient effort, guiding students through coping mechanisms in difficult situations, encouraging social persuasion, and using similar ability (learning needs) peers as models. These sources of mathematics self-efficacy are embedded in the teacher training to provide an effective intervention for enhancing the construct.

### Learning-based interventions

These are interventions that foster mathematics self-efficacy through manipulations of students' learning strategies. An effective intervention in this category is the integration of four self-efficacy features – anxiety coping strategy (affective states), modeling example (vicarious experience), mental practice (mastery experience), and effort feedback (social persuasion) – into a computerized example-based learning activity. For instance, Ramdass and Zimmerman (2008) report an intervention study in which a step-by-step solution strategy was supplemented with self-correcting strategy for improved mathematics self-efficacy. Students in the experimental group are trained on using some strategies to check whether their answers are correct in addition to the step-by-step solution method. The intervention proves effective, and its underlying mechanism lies in using mastery experience coupled with self-persuasion to foster mathematics self-efficacy.

Akin to the manipulation of learning strategy as a proxy to foster students' mathematics self-efficacy is computerized example-based intervention by Huang et al. (2020). They created a computerized learning environment that students used to learn and practice some statistical skills after following some worked examples. Students in the integrated example-based treatment condition started the experiment by listening to some anxiety coping strategies disguised as instructions. Then, they followed some worked examples that are presented by an animated expert to provide vicarious experience. At the end of each model example, students engaged in some mental practices to provide mastery experience. Students then proceed to solve their presented questions at the end of which are some feedback statements that provide social persuasion to the students. A sample feedback statement is “Your answer is not 100% correct. Don't give up. Focus on the next example-problem pair. Study the example carefully. With hard work, your performance will improve” (Huang et al., 2020, p. 1018). The basic mechanisms that foster mathematics self-efficacy in this intervention are the four sources of self-efficacy embedded in learning strategy.

### Interventions with the highest effect on self-efficacy

To address the research question two of this review, I take a closer look at Table 1 and argue for the intervention with the highest effect on self-efficacy among the presented nine intervention studies. At this stage, three out of the nine studies are screened out from the discussion that follows because their authors do not explicitly report the effect sizes of the interventions (Ramdass and Zimmerman, 2008; Kandil and Işiksal-Bostan, 2019; Kohen et al., 2019). Half of the remaining six studies focus on pre-university students: primary school students with age ranging from 7 to 9 years (Siegle and McCoach, 2007; Bonne and Johnston, 2016), and lower secondary school students with age ranging from 13 to 14 years (Brisson et al., 2017). The other half of the studies focus on university students (Luzzo et al., 1999; Cordero et al., 2010; Huang et al., 2020). As such, if the focus is to improve pre-university students' mathematics self-efficacy then the intervention (self-efficacy features embedded in a teacher training) by Siegle and McCoach (2007) is expected to have the highest impact on mathematics self-efficacy. The reported effect size is 0.46 for an intervention that lasted for 4 weeks. Interestingly, a step-by-step implementation of this intervention including resources such as videos and teachers' training notes are freely available online (https://nrcgt.uconn.edu/underachievement_study/self-efficacy/SE_Section0/). In the implementation of this recommendation, one should consider the age of participants, the country in which the research was conducted and the associated cultural factors. Moreover, Siegle and McCoach (2007) did not report the sustained effect of their intervention which is a crucial factor that should be considered in the implementation of this recommendation. For instance, the effect size of the intervention by Brisson et al. (2017) is smaller than 0.46 but evidence shows that the effect was sustained for more than 6 weeks. It would have been more interesting if such enduring effect of the interventions is reported by Siegle and McCoach (2007).

On the other hand, if the focus is to improve university students' mathematics self-efficacy then the intervention (computerized example-based learning equipped with self-efficacy features) by Huang et al. (2020) is expected to have the highest impact on mathematics self-efficacy. The reported effect size is 0.71 for an intervention that lasted for 1 h. However, the enduring period of the intervention effect size by Huang et al. (2020) is not reported. Unlike the self-efficacy intervention reported by Luzzo et al. (1999) which has an effect size of 0.51 with an enduring effect of up to 6 weeks. As such, one may favor the intervention by Luzzo et al. (1999) over the one by Huang et al. (2020) if both effect sizes and the enduring effects of the interventions are considered during implementation. It is acknowledged that recommending interventions based on effect sizes could be naïve. The problem here is that different measures of the effect size have been used in different studies and none of them is absolute in the sense that they would allow an absolute comparison of the effectiveness between different studies (Bakker et al., 2019; Simpson, 2019). Following the line of thought by Simpson (2019), a higher Cohen's d in Study 1 than in Study 2 does not necessarily mean a larger effect in Study 1 if different measures of mathematics self-efficacy have been used in these studies. Moreover, different intervention studies contained in this review are based on interventions with varying durations which may affect the interpretation of the effect sizes (Bakker et al., 2019). It is a challenge to determine, how comparable are effects measured after “a 10-min presentation” or after “3-month intervention”. Finally, if an intervention made in primary schools gives the same effect as another intervention made in tertiary institutions, can one say that the interventions are equally effective? I would interpret that the latter has made a more significant effect because of the more demanding starting point for the intervention. In sum, all the nine interventions are crucial to improving mathematics self-efficacy. This section only provides additional information for those who are interested in the quantification of the impacts of such interventions.

## Conclusion

To conclude, mathematics self-efficacy is an important factor that has been widely investigated among researchers on affect in mathematics education research. It plays crucial roles in predicting students' success in mathematics and other cognitive and affect factors. This research reports a systematic review of studies that demonstrate experimentally some strategies to increase students' mathematics self-efficacy. Some effective interventions are identified and described including their underlying mechanisms that foster self-efficacy. Three important groups are identified for the categorization of self-efficacy interventions. First, interventions that directly manipulate sources of mathematics self-efficacy (e.g., Luzzo et al., 1999; Cordero et al., 2010). For instance, an intervention that provides vicarious experience to students by showing them a video presentation of an older student that narrates her/his experience including coping mechanism while following the same course (Luzzo et al., 1999). Second, are the instructional-based interventions. This category of interventions embed some sources of self-efficacy into teaching methods or teacher training programs (e.g., Bonne and Johnston, 2016; Kandil and Işiksal-Bostan, 2019). An example of interventions in this category is the teaching method that combined mastery experience with social persuasion to foster students' mathematics self-efficacy (Bonne and Johnston, 2016). The third theme comprises interventions that embed self-efficacy features in learning activity of the students. For example, the computerized example-based learning with specially designed features for improved self-efficacy is in this category (Huang and Mayer, 2018).

Moreover, I also identified two most effective interventions in fostering mathematics self-efficacy. These two interventions are reported by Siegle and McCoach (2007) for pre-university students and by Huang et al. (2020) for university students. By implication, the I recommend the reported interventions in these two studies for improved mathematics self-efficacy among pre-university and university students. It is envisaged that if these interventions are implemented the effect will transcend students' convictions about their mathematical capability to improved performance in mathematics. However, one should put into consideration factors such as age and gender of the participants, year of students' study, and cultural diversity of the country. As such, the researcher also recommends replications of these interventions in independent students' populations.

It is crucial to remark that most of the reviewed studies reported the effect sizes of the self-efficacy interventions but very few studies (only four) reported the enduring effect of the respective interventions. This lack of clarity on the enduring effect of mathematics self-efficacy interventions is a limitation of previous intervention studies that future studies should intend to address. The researcher acknowledges that incorporating enduring effect into the design of intervention studies is demanding. Perhaps, the difficulty involved in the design of such studies is the reason why most of the previous intervention studies did not report on the enduring effect. However, a total reliance on the effect size to judge the effectiveness of a self-efficacy intervention is risky. This is because an intervention may have a large immediate effect that fades away soon afterwards. On the contrary, a small immediate effect of an intervention may be sustained for a long time. Therefore, I recommend an adequate attention to the enduring effect in the design of further self-efficacy intervention studies. Further, classroom teachers and instructors may consider using multiple interventions to complement each other such that the students' self-efficacy may improve.

## Data availability statement

The raw data supporting the conclusions of this article will be made available by the authors, without undue reservation.

## Author contributions

YZ: conceptualization, methodology, formal analysis, software, data curation, investigation, visualization, and writing-original draft preparation.

## Conflict of interest

The author declares that the research was conducted in the absence of any commercial or financial relationships that could be construed as a potential conflict of interest.

## Publisher's note

All claims expressed in this article are solely those of the authors and do not necessarily represent those of their affiliated organizations, or those of the publisher, the editors and the reviewers. Any product that may be evaluated in this article, or claim that may be made by its manufacturer, is not guaranteed or endorsed by the publisher.
